# Italian healthcare professionals’ role in advancing reforms within the Italian National Healthcare System, as outlined in the National Recovery and Resilience Plan

**DOI:** 10.3389/fpubh.2025.1603708

**Published:** 2025-07-01

**Authors:** Martina Giusti, Alessandro Beux, Teresa Calandra, Stefano Lorusso, Paolo Amilcare Gazzaniga, Guglielmo Bonaccorsi, Giuseppe Greco, Niccolo Persiani

**Affiliations:** ^1^Department of Experimental and Clinical Medicine, University of Florence, Florence, Italy; ^2^Centro Studi SAPIS Foundation, Italian National Federation of Orders of Radiographers and Technical, Rehabilitation, and Prevention Health Professions Research Centre, Rome, Italy; ^3^Azienda Ospedaliero Universitaria Città della Salute e della Scienza, Turin, Italy; ^4^Azienda Sanitaria Locale di Colegno e Pinerolo, Colegno, Turin, Italy; ^5^Ministry of Health, Rome, Italy; ^6^MEDGLOX, Ivrea, Italy; ^7^Department of Health Sciences, University of Florence, Florence, Italy; ^8^School of Human Sciences, University of Florence, Florence, Italy

**Keywords:** Italy, national healthcare system, reform, allied health professionals, policymaker decision support system

## Abstract

**Introduction:**

In 2021, the National Recovery and Resilience Plan (PNRR) outlined the foundational pillars for Italy’s economic recovery following the COVID-19 pandemic. Within PNRR’s Mission 6 “Health”, guidelines were established to reform the Italian healthcare system, focusing on community-based services and digitalization. This ambitious reform aims to shift from a hospital-centred model to a patient-centred one that promotes integrated provision of social and healthcare services.

**Methods:**

The lack of institutional working groups to incorporate input from all Italian recognized healthcare professionals into PNRR’s implementation, the National Federation of Orders of Radiographers and Technical, Rehabilitative, and Prevention Health Professionals (FNO TSRM & PSTRP) addressed this gap by initiating a study.

**Results:**

The contributions from the 19 Italian Allied Health Professionals (AHPs) affiliated with the FNO TSRM & PSTRP were collected to identify priority areas and formulate actionable recommendations aimed at enhancing the organizational model of reformed Italian healthcare system outlined by the PNRR, which had been predominantly focused on nursing care.

**Discussion:**

Adopting a multi-professional and multidisciplinary approach could better address social and health needs of the population by leveraging the expertise of all registered Italian health professionals. This approach aligns Italian healthcare system with European standards, addressing issues such as task shifting and shortages in professional resources. Ultimately, this study provides concrete insights for Italian policymakers and healthcare leaders to foster greater inclusion of Italian AHPs in the design and implementation of the current and future reforms on national healthcare system.

## 1 Introduction

Healthcare systems worldwide are undergoing significant reforms to address a range of complex and interconnected challenges. Among the most pressing issues are the rising costs of medical care, which strain public and private funding sources; the demographic shift toward aging populations, which increases the demand for long-term care and age-related health services; and the increasing prevalence of chronic diseases, such as diabetes, cardiovascular conditions, and respiratory illnesses. These factors not only place substantial pressure on existing healthcare infrastructure and workforce capacity but also call for innovative strategies in policy, technology adoption, and care delivery models to ensure sustainable, equitable, and high-quality healthcare for all (1). A key point of these reforms is transitioning from hospital-centered care to patient-centered care, emphasizing integrated social and healthcare services delivered closer to patients to ensure continuity of care (2). Allied health professionals (AHPs)—a diverse group encompassing diagnostic, rehabilitative, and prevention-focused health professionals—play a critical role in achieving these goals (3, 4). Despite their essential contributions to healthcare delivery, AHPs remain underrepresented in shaping and implementing system-wide reforms (5, 6). Historically, reforms have emphasized multiprofessional collaboration but have largely relied on working groups composed mainly of physicians and nurses (7, 8).

Evidence increasingly highlights AHPs’ unique ability to bridge gaps in care delivery and drive innovation in health service provision (9). For example, expanding AHPs’ scope of practice in primary care settings has been shown to reduce physician workload, improve patient access to services, and enhance health outcomes (10, 11). However, systemic barriers—including limited representation in policymaking and restrictive regulatory frameworks—continue to hinder their active participation in reform initiatives (12, 13).

The Italian healthcare system is renowned for its universal access and regionalized governance but faces growing challenges due to demographic shifts, financial constraints, and the burden of chronic diseases (14). Recent reforms have emphasized integrated care models, patient-centered services, and interprofessional collaboration to improve efficiency and equity across the system. AHPs are particularly well-positioned to contribute to these efforts by managing chronic diseases and supporting rehabilitation initiatives through their specialized knowledge and expertise (15).

They may specifically complement the existing understanding of the Italian healthcare system, which is primarily informed by the perspectives of physicians and nurses. While these viewpoints are undoubtedly valuable, they do not fully capture the complexity and multidimensionality of healthcare delivery. AHPs’, due to their specialized competencies and holistic approach to patient care, offer unique insights that could enrich the system’s responsiveness, efficiency, and patient-centeredness. Despite this potential, their involvement in shaping and implementing healthcare reforms in Italy remains relatively limited, both in practice and in policy discourse. Enhancing their engagement could lead to more inclusive, interdisciplinary approaches that better reflect the diverse needs of the population and promote more sustainable healthcare transformation (16, 17).

Italian healthcare reforms have increasingly focused on regional autonomy, leading to variations in how services are delivered and how AHPs are integrated into care models (18). However, systematic and programmatic approaches for employing AHPs within new community-based health facilities established by Mission 6 “Health” of the National Plan for Recovery and Resilience are still lacking (19, 20) despite AHPs’ potential contributions. Their specific competencies enable them to:

- enforce multiprofessional working groups in primary healthcare (13)
- drive digitalization processes (21),
- personalize clinical care pathways (22)
- readapt protocols and procedures according to emerging organizational needs and/or assisted person’s needs (6)
- conduct research (12, 23) and more.

In Italy, the insufficient representation of AHPs in decision-making bodies hinders their active involvement in reform initiatives (24, 25).

To harness the potential of AHPs in transforming healthcare systems globally, this study aims to explore the unique contributions of Italian AHPs to the ongoing reform of the Italian healthcare system. These professionals represent 19 recognized health professionals involved in diagnosis, assistance, rehabilitation, and prevention. By examining their roles, expertise, and experiences, the objective is to inform the development of more inclusive and responsive health policies, foster innovation in service delivery models, and strengthen workforce capacity across the country. These efforts are analyzed in relation to the structural opportunities, systemic barriers, professional specialties, and persistent challenges within the Italian healthcare system. By synthesizing current evidence and best practices, this study offers actionable insights for policymakers, healthcare leaders, and AHPs to promote greater inclusion of AHPs in shaping the future of healthcare in Italy.

## 2 Materials and methods

For the objectives outlined, a qualitative methodology was selected to best explore the engagement of allied health professionals within the reformed Italian healthcare system (1–3). The research was conducted following the study protocol described below.

**Figure 1:**
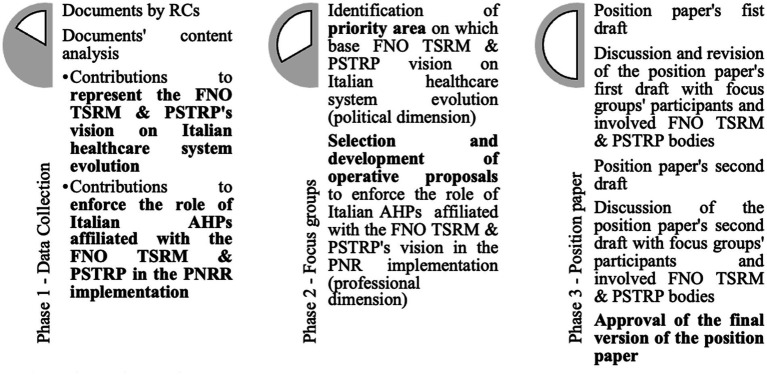
Phases of research protocol.

In the first instance, the research group started a collaboration with the FNO TRSM & PSTRP, institutional representation of the Italian 220,000 registered professionals involved in the diagnosis, assistance, rehabilitation, and prevention areas. According to the Italian model of registered health professionals, the National Federation of Orders of Radiographers and Technical, Rehabilitative, and Preventive Health Professional (FNO TSRM & PSTRP) has a complex organization composed of many different sub-organizations. Among these, the first interlocution was with the Central Committee (CC) of the FNO TSRM & PSTRP, the political body of the FNO TSRM & PSTRP responsible for coordination, representation, and protection of the health professionals. It also plays a coordinating role over the provincial and interprovincial Orders of TSRM and PSTRP. In addition, the CC is committed to promoting the exchange of ideas and resources, including with institutions and civil society. The CC of the Federation has made itself available to put the research group in contact with the national council (NC) and registered commissions (RCs) of the 19 health professionals within the FNO TRSM & PSTRP (25) (Figure 1). The NC is the government body of the FNO TRSM & PSTRP, composed of local Orders’ presidents and has the tasks of (a) electing the members of the CC and the Board of Auditors; (b) approving the budget and the final account of the FNO TRSM & PSTRP, included extraordinary expenditures and annual contribution that each Order must pay for the operating expenses of the FNO TRSM & PSTRP; and (c) approving the internal regulations of the FNO TRSM & PSTRP. The 19 RCs are, instead, the professional bodies in the FNO TSRM e PSTRP, one for each professional. These are responsible (a) to propose to the NC the registration of new applying professionals; (b) to adopt and execute disciplinary measures against all registered members and all other disciplinary and sanctioning provisions contained in the laws and regulations in force; (c) to exercise the management functions included in its competences; and (d) to give its assistance to the local authorities in the study and implementation of measures that may, in any case, affect the represented professionals. To which one of these three bodies was it asked to design members for the following focus groups?

### 2.1 Phase 1 — Data collection

After conducting a literature review to investigate the role of technical, rehabilitation, and prevention health professionals internationally, the Central Committee of the FNO TSRM & PSTRP requested that the RCs develop a concise document (no more than 3,000 words). This document aimed to identify the potential contributions of FNO TSRM & PSTRP health professionals to the evolution of the Italian National Health System (NHS) as initiated by the PNRR and to put forward operational proposals for implementing the PNRR itself. The proposals were to enhance the role of the FNO TSRM & PSTRP health professionals within multiprofessional and multidisciplinary working groups, both in traditional care settings and, especially, in the new territorial structures envisioned by the PNRR (such as community homes, community hospitals, territorial operations centeres, and patients’ homes). Once the documents were received from all RCs, the working group systematized the contributions related to the FNO TRSM & PSTRP’s vision for the evolution of the NHS based on a content analysis of these texts (26). The results of the content analysis were then discussed in a cycle of focus groups with members of the Federation’s Central Committee, the presidents of the RCs, and the presidents of the Orders. Concurrently, the research group listed the operational proposals put forward by the individual RCs, integrated similar proposals, and organized them according to the articulation of the PNRR so that they could substantively contribute to the organizational model presented within it. The identified proposals were further discussed and refined in a working group that included professionals without any formal role in the order structure.

### 2.2 Phase 2 — Focus groups

The information derived from the RCs’ documents was discussed in two separate cycles of three focus groups each (27, 28). Each online meeting, lasting approximately 2 h, was held 1 week after the previous one, following the distribution of preparatory material to the participants at least 2 days in advance.

The results of the content analysis were discussed in focus groups attended by designated members from the CC, RCs, and NC to outline the FNO TSRM & PSTRP’s vision for the evolution of the NHS. These individuals were specifically involved as representatives of the Federation’s political dimension, capable of developing the Federation’s vision for the evolution of the Italian National Healthcare System (29). The group consisted of 24 people, with eight from each target group (Table 1). The systematization of the operational proposals put forward by the individual RCs was discussed in focus groups attended by health professionals identified by the Central Committee of the Federation, RCs, and NCs but not belonging to them. This ensured that the professionals involved could contribute with their knowledge, skills, and professional experience (30, 31). This group was composed of 30 people (Table 2). Prior to the two focus group cycles, a preliminary online meeting was held to explain the purpose of the project and to obtain informed consent from the participants. The members of the research group divided their time between the two focus groups, cyclically playing the roles of leaders and observers.

**Table 1:** Health professionals involved in the focus group on “the FNO TSRM & PSTRP vision on Italian healthcare system evolution”.

	Central committee (CC)	Registered commissions (RCs)	National council (NC)
Involved health professionals	Member 1 (Radiographer)	Member 9 (Radiographer)	Member 17 (Radiographer)
	Member 2 (Radiographer)	Member 10 (Radiographer)	Member 18 (Radiographer)
	Member 3 (Dental Hygienist)	Member 11 (Biomedical laboratory technician)	Member 19 (Professional educator)
	Member 4 (Podiatrist)	Member 12 (Orthopaedic Technician)	Member 20 (Biomedical laboratory technician)
	Member 5 (Orthoptist)	Member 13 (Health Care Assistant)	Member 21 (Child neuro and psychomotor therapist)
	Member 6 (Prevention technician in the environment and in the workplace)	Member 14 (Radiographer)	Member 22 (Prevention technician in the environment and in the workplace)
	Member 7 (Occupational therapist)	Member 15 (Hearing Care Technician)	Member 23 (Radiographer)
	Member 8 (Prevention technician in the environment and in the workplace)	Member 16 (Biomedical laboratory technician)	Member 24 (Radiographer)

**Table 2:** Health professionals involved in the focus group on “the role of health professionals belonging to FNO TSRM & PSTRP in reformed Italian healthcare system”.

	Rethinking PHC in community homes	Strategic reprogramming of the hospital technology and digital park	Enhancement of home teleassistance	Rethinking PHC in the community hospital
Involved health professionals	Member 1 (Dietician)	Member 10 (Audiometric Technician)	Member 16 (Techniques of cardiovascular pathophysiology and cardiovascular perfusion)	Member 24 (Psychiatric Rehabilitation Technician)
	Member 2 (Dietician)	Member 11 (Radiographer)	Member 17 (Techniques of cardiovascular pathophysiology and cardiovascular perfusion)	Member 25 (Speech therapist)
	Member 3 (Orthoptist)	Member 12 (Neurophysiopathologist Technician)	Member 18 (Health Care Assistant)	Member 26 (Hearing Care Technician)
	Member 4 (Speech therapist)	Member 13 (Biomedical laboratory technician)	Member 19 (Psychiatric Rehabilitation Technician)	Member 27 (Dental Hygienist)
	Member 5 (Speech therapist)	Member 14 (Radiographer)	Member 20 (Biomedical laboratory technician)	Member 28 (Occupational therapist)
	Member 6 (Dental Hygienist)	Member 15 (Radiographer)	Member 21 (Orthopaedic technician)	Member 29 (Child neuro and psychomotor therapist)
	Member 7 (Professional educator)		Member 22 (Dental Hygienist)	Member 30 (Health Care Assistant)
	Member 8 (Child neuro and psychomotor therapist)		Member 23 (Occupational therapist)	
	Member 9 (Occupational therapist)			

#### 2.2.1 Focus group on “The FNO TSRM & PSTRP vision of Italian healthcare system evolution”

In preparation for the first focus group, the results of the content analysis were shared with the participants for their review. During this focus group, the group identified which elements of the content analysis could be used to construct the FNO’s vision of the evolution of the NHS. The coding research group extracted points from the focus group transcript that were mentioned as possible bases for articulating the Federation’s vision and shared them with the participants. In the second focus group, the group identified 10 of these results. The research group then developed a brief description of each, based on the discussions during the second focus group, and made these descriptions available to the participants. During the third and final focus group, the participants reviewed the descriptions and ordered them in such a way as to represent the framework from which the research group was tasked with formulating the draft summary. This summary would form part of the position paper by the FNO on “The relaunch of the Italian national healthcare service. Proposals for the implementation of the National Recovery and Resilience Plan. The contribution of the FNO TSRM & PSTRP’s 19 professionals.”

#### 2.2.2 Focus group on “The role of health professionals affiliated with to FNO TSRM & PSTRP in reformed Italian healthcare system”

In preparation for the first focus group, the operational proposals put forward by the Boards of Directors were shared with the participants. Starting from this list of proposals, the participants validated the organization of the proposed projects in relation to the structure of the PNRR, believing that this would be useful for understanding the role of health professionals in the new NHS. They then selected the projects considered to be of greatest interest. In preparation for the next focus group, the research team extracted areas of common interest among the participants by coding the focus group transcript and sharing these with the participants.

In the second focus group, the participants discussed how to enhance these areas of common interest and decided to develop four multiprofessional and multidisciplinary projects, starting from the areas of common interest previously identified. The group was then divided into four subgroups that began to develop a first draft of the projects, which were concluded during the third focus group. At the end of this process, the participants tasked the research group with developing a first draft corresponding to the second part of the position paper by the FNO, based on the selected design lines and developments.

### 2.3 Phase 3 — Position paper

All members of the CC, NC, and all 19 presidents of each RC, as well as those who participated in the focus groups but were not part of any of the previous categories, were invited to the presentation (online) of the draft of the position paper for giving their opinion on that. This draft was developed by the research group summarizing the contributions from the two working groups. Ten days before the presentation, the first draft of the position paper was shared via email with all participants, who were asked to provide any observations within 3 days. One week before the meeting, a second version of the draft, including any received suggestions, was sent. During the online meeting, this second version was discussed. The formal nature of the final suggestions allowed the validation of the draft to proceed, thereby creating the final version of the position paper despite the opportunity to make extemporaneous observations during the meeting.

## 3 Results and discussion

Drawing on theoretical frameworks, such as interprofessional collaboration and organizational rethinking of the healthcare workforce, the involvement of allied health professionals in the reform of the Italian healthcare system was explored. Below, the two sections of the FNO TSRM & PSTRP position document are discussed distinctly to examine the roles of the 19 health professionals represented by this Federation in policy development, service delivery innovation, and workforce capacity building, with a focus on the challenges and opportunities unique to the Italian system.

### 3.1 Findings from a focus group on “the FNO TSRM & PSTRP vision of Italian healthcare system evolution”

The first section of the position paper by *FNO TSRM & PSTRP* highlights the strategy for effectively implementing the Italian NHS reform, emphasizing the essential role of allied health professionals across 10 key points (Table 3).

**Table 3:** Key points identified by the focus group on “the FNO TSRM & PSTRP vision of Italian healthcare system evolution”.

Key points	
I	Creation of national and local hospital networks for specialization and technological innovation
II	Task shifting and empowerment of health professionals in multidisciplinary working group
III	Health, environment, and climate protection as a principle of sustainable development
IV	Promoting individual and collective health in daily life and workplace settings
V	Integrated care pathways through multidisciplinary working group
VI	Strengthening community and home-based care through digital health
VII	Modernizing essential levels of care and streamlining service delivery
VIII	Investing in academic training to combat professional illegitimacy
IX	Fostering lifelong learning and continuous professional development
X	Research as a driver of the healthcare system and professional development

#### 3.1.1 Creation of national and local hospital networks for specialization and technological innovation

The Federation designates the hospital as a central reference point for managing complex cases requiring highly specialized care, which cannot be comprehensively provided in community-based health facilities.

This approach aligns with the proposals outlined in Mission 6 “Health” of the PNRR, which recognizes the hospital’s strategic role as a hub for high technology and short-stay hospitalization (16). Complex patients are assessed here before being directed to care pathways in facilities of lower complexity and closer to their homes following the resolution of the acute phase (7, 32). To do that, the creation of national and local hospital networks for specialization and technological innovation is mandatory. University hospitals and IRCCCS^1^ serve as engines in the Italian healthcare systems as promoters of organizational, technological, educational, and professional innovation through scientific research (33–36).

#### 3.1.2 Task shifting and empowerment of health professionals in multidisciplinary working group

Competencies in healthcare cannot be exercised within a siloed system where professionals work in isolation and claim exclusivity over their tasks. Multidisciplinary integration is necessary to best meet the evolving needs of citizens. The future organizational model of the Italian healthcare system must experiment with the joint and shared actions of healthcare professionals working in teams, overcoming rigid constraints among recognized Italian health professionals (37, 38). This requires a continuous reconsideration of the tasks, roles, and competencies of healthcare professionals based on the principle that healthcare actions (interventions, services) should be performed by those who are most capable of carrying them out. The team decides and assigns specific professional roles to a professional based on the possessed certified and applied competencies, and the designated professional assumes the full responsibility (including civil and criminal liability) for fulfilling the own tasks.

#### 3.1.3 Health, environment, and climate protection as a principle of sustainable development

Health, environment, and climate protection must inherently consider the respect of the field of action, ensuring its sustainable development over time (39). The adoption of Health Technology Assessment (40) and Health Impact Assessment (41) approaches can help promote the accountability of sustainability within healthcare organizations. To further disseminate these approaches, specific training should be promoted for professionals within the Federation, taking into account their existing knowledge and skills on the topics (42).

#### 3.1.4 Promoting individual and collective health in daily life and workplace settings

The need to ensure the promotion of individual and collective health in daily life and workplace settings, directly addressing risk factors and health determinants, is as equitable as possible if adequate resources for tailored campaigns, especially for socioeconomically vulnerable groups, are invested in these (43). In this way, it is remarked on the importance of upholding the fundamental principles of the Italian National Health Service: universality, equity, and equality. Additionally, the Federation highlights a distinctive Italian excellence, specifically the recognition of two health professionals designed for disease prevention and health promotion: the health care assistant and the prevention technician in the environment and in the workplace. Prevention departments, identified as the coordination centers for the proposed interventions, position these professionals at the forefront (4).

#### 3.1.5 Integrated care pathways through multidisciplinary working group

The Federation promotes the establishment of integrated care pathways through multidisciplinary working group, complementing GPs, who are usually overloaded (44), to jointly enforce and enlarge the healthcare services provision for the entire population within the new community-based health facilities planned in the reformed Italian National Health Service (6, 7). This approach would improve the effectiveness of the service provided through the personalization of care pathways. From the Federation’s perspective, the reform process of the Italian SSN should culturally lead to the establishment of Health Literate Healthcare Organizations (45, 46).

#### 3.1.6 Strengthening community and home-based care through digital health

The enhancement of community and home-based care within the Italian healthcare system is supported by investments in digital systems, which serve as key enablers for integrated and personalized care for the entire population—especially for chronic, frail, and non-self-sufficient patients—across the continuum of healthcare, social-healthcare, and social-assistance services (17, 47). The ongoing reform process should not be understood merely as the digitization of current analogic systems but, above all, as an opportunity for rethinking operational models and interactions among all stakeholders involved (25, 48). Digital technologies are, therefore, a useful but not sufficient prerequisite for the evolution of modern healthcare systems (49, 50).

The availability of technological tools must necessarily be accompanied by:

- The identification of new professional opportunities for AHPs, leveraging the knowledge and skills acquired during their specialization (for example, as system administrators of the various digital platforms that, by integrating, create both an informational and operational network).
- The initiation of new training programs for professionals so that they can take on roles as trainers, facilitators, guides, and consultants for the final users of digital healthcare and telemedicine tools—namely, citizens, from young adults to older adults.

Without such targeted actions, the intended goals will not be met due to persistent knowledge and cultural barriers.

#### 3.1.7 Modernizing essential levels of care and streamlining service delivery

The provision of digital and remote medicine services also requires the modernization of essential levels of care and the streamline of health service delivery to make healthcare services effectively accessible and usable. This is particularly important for services not yet formally codified for remote medicine, such as diagnostic and rehabilitative services. If the ongoing reform of the Italian healthcare system aims to strengthen integrated care for the entire population, it is essential to overcome bureaucratic barriers that currently separate the management of healthcare services from social ones. The existence of two distinct coding lists for healthcare services (51, 52) and social services (53, 54), with partial overlap in the case integrated care, is outdated and ineffective.

#### 3.1.8 Investing in academic training to combat professional illegitimacy

Investing in university education is a key strategy to combat academic malpractice and prevent professional illegitimacy, both of which pose serious threats to the quality of care and public health. In this context, strengthening higher education is essential—not only in terms of curriculum content but also by increasing capacity through the expansion of bachelor’s and master’s degree programs, postgraduate diplomas, advanced specialization courses, and PhD pathways (55, 56). These efforts would help address workforce shortages in healthcare and foster a stronger alignment between academic training and labor market needs. The true strength of university education lies in its ability to deliver comprehensive, interdisciplinary training that integrates theoretical knowledge with practical application. This equips future professionals to enter the workforce with confidence and encourages them to actively and responsibly apply their expertise in real-world contexts.

#### 3.1.9 Fostering lifelong learning and continuous professional development

Fostering lifelong learning and continuous professional development are the foundations for professional growth, which means not only enhancing the skills of individual practitioners but also enabling a professional to adapt its work overtime—with tenacity and resilience—to the ever-evolving health needs of citizens (57). Given the increasing complexity of healthcare systems, specific managerial and technical-specialist training programs tailored to various professional dimensions would be a valuable resource to support the continuous growth of the system. When approached as a continuous process of improvement and optimization, the development of managerial and technical-specialist skills among AHPs can significantly enhance their role in the multidisciplinary delivery of health promotion and patient care. At the same time, it enables a more qualified and effective response to the evolving needs of healthcare organizations (48).

#### 3.1.10 Research as a driver of healthcare system and professional development

All this cannot be achieved without considering research as a driver of the healthcare system and professional development. The professional practice of every healthcare worker must be guided by evidence derived from clinical research. Healthcare professionals must be actively engaged in research projects, thereby advancing translational medicine and contributing to the collective expertise of the scientific community to which they belong (58). Additionally, the establishment of multiprofessional and multidisciplinary research groups within various settings—whether in community-based facilities, hospitals, or in collaboration with municipalities, NGOs, and industries [pharmaceutical, biomedical, and information and communication technology (ICT)]—enables the development of innovative studies (59, 60). However, human resources (in terms of health professionals) policies in Italian healthcare organizations should be reformed to encourage health professionals to conduct research. Scientific Institute for Research, Hospitalization and Healthcare (IRCCS), university hospitals, and tertiary-level hospitals within Local Health Authorities should be examples because, in these institutions, research and innovation are strategic priorities (61, 62).

### 3.2 Findings from a focus group on “the role of health professionals belonging to FNO TSRM & PSTRP in reformed Italian healthcare system”

The second section presents 27 project lines (Table 4), each aligned with specific components, investments, and reforms enacted by Mission 6 “Health” of the PNRR (16). These project lines were developed to properly implement the PNRR.

**Table 4:** Project lines identified by a focus group on “the role of health professionals belonging to FNO TSRM & PSTRP in reformed Italian healthcare system”.

Project lines
PL 1: Establishment of a multidisciplinary and multiprofessional ministerial working group to define minimum team composition for Community House
PL 2: Update of the Essential Levels of Care (LEA), social services, and the Outpatient Specialty Nomenclature.
PL 3: Promotion of healthy lifestyles and primary prevention through a national “Health Literacy Empowerment” campaign targeting patients and caregivers.
PL 4: Implementation of screening programs for diseases impacting survival, quality of life, and individual autonomy.
PL 5: Initiatives to promote behaviors that enhance wellbeing, quality of life, self-determination, and autonomy.
PL 6: Development of training and support programs for parenting skills, especially for families in vulnerable situations.
PL 7: Integration of data collection in the care processes within Community Houses.
PL 8: Activation of specialized units to ensure continuity of care across settings.
PL 9: Strengthening and revitalization of community-based social and health facilities, drawing from existing best practices.
PL 10: Remote health monitoring for individuals at home and workers in their workplace.
PL 11: Delivery of diagnostic services at home or in proximity through mobile multiprofessional units targeting chronic and frail patients.
PL 12: Integration of home-based care services with remote consultations and monitoring, feeding into the national Electronic Health Record (EHR) system.
PL 13: Enhancement of rehabilitation services to foster self-sufficiency and social inclusion
PL 14: Working group focused on defining the minimum multiprofessional team for Community Hospitals.
PL 15: Implementation of second-level diagnostic units within Community Hospitals.
PL 16: Creation of networks of excellence integrating professional, technological, and organizational expertise.
PL 17: FNO TSRM & PSTRP’s involvement in supporting decision-makers in healthcare investment planning.
PL 18: Testing of innovative technologies via “managed-entry agreements” with manufacturers.
PL 19: Optimization of the hub-and-spoke model for equitable distribution of medical technologies between hospitals and community facilities.
PL 20: Development of eco-sustainable hospitals with energy autonomy, waste reduction, and modular, flexible spatial design.
PL 21: Investment in programs to prevent Healthcare-Associated Infections (HAIs).
PL 22: Training programs on clinical risk management for healthcare personnel.
PL 23: Physical and psychological health surveillance for healthcare professionals.
PL 24: Investment in database integration to establish a unified Clinical Repository of Big Data.
PL 25: Establishment of the FNO TSRM & PSTRP Research Center.
PL 26: Training programs to enhance healthcare professionals’ digital skills.
PL 27: Training programs to strengthen healthcare professionals’ managerial and organizational competencies.

#### 3.2.1 M6C1 Networks of proximity, structures, and remote medicine for community-based care

As part of Component 1 of Mission 6 of the PNRR, titled “Networks of proximity, structures, and telemedicine for territorial healthcare,” Italian AHPs have proposed the establishment of a multidisciplinary and multiprofessional ministerial working group to support the implementation of Investment 1.1: “Community Houses and Person-Centered Care.” The primary objective is to define the core composition of the minimum multiprofessional team that must be constantly present in each Community House. Concurrently, it is important to expand the essential levels of care, the essential level of social services, and the nomenclature for outpatient specialist care to ensure the full availability of services required to meet citizens’ health needs (51, 53). Community houses should serve as a primary point of reference for the population, particularly in the following areas (43):

- Primary prevention, including health promotion campaigns and education activities for patients and caregivers, aimed at empowering citizens (PL 3).
- Secondary prevention, through the implementation and expansion of screening programs (PL 4), is not only for early diagnosis of life-threatening diseases but also for promoting behaviors that impact quality of life and personal autonomy (PL 5).

The Community House is envisioned as a physical and accessible hub where individuals can connect with the healthcare system and receive comprehensive responses to their health needs (20). Within this setting, programs supporting parenting skills and vulnerable families and children in (PL 6) can be developed in collaboration with third-sector organizations and volunteer associations. These initiatives should also foster a culture of data awareness among healthcare professionals and users, strengthening health-related information flows (PL 7 and PL 24). For individuals in fragile conditions, the home becomes the primary setting for territorial assistance (Investment 1.2). In the early stages of designing and validating care pathways in this “new” setting, it is crucial to deploy Specialized Continuity of Care Units (USCA) to promptly ensure responses (PL 8). Additionally, successful remote medicine practices should be evaluated using objective data and integrated into the standard offerings of the National Health Service (NHS), becoming a structural element of the country’s digital health strategy (PL 9). These efforts lay the groundwork for remote health monitoring projects, both at home and in workplaces, enabling preventive and corrective actions to safeguard individual and public health (PL 10 and PL 12). This includes conducting diagnostic tests directly at home (PL 11) and enhancing rehabilitation services to support autonomy and social inclusion—through home-based rehabilitation visits, remote evaluation, and remote rehabilitation (PL 13). The expansion of home radiology and screening services, including those offered at Community Houses, is also encouraged, given the average complexity of patients’ health conditions in these settings (PL 15) (63).

On the other hand, concerning the Community Hospital (35), it may be appropriate to organize a dedicated unit for second-level diagnostics (e.g., CT scans and advanced neurological monitoring), ensuring that patients are referred to hub hospitals for third-level care only when necessary.

Finally, the creation of an institutional, multidisciplinary, and multiprofessional working group is essential to express an opinion on the composition of the minimum multiprofessional team (8, 64). Each care setting must consistently include such a team, which can be expanded as needed based on individual cases, including the involvement of AHPs (PL 14).

#### 3.2.2 M6C2 innovation, research, and digitalization of the national healthcare system

In the reorganization of the IRCCS (Scientific Institutes for Research, Hospitalization, and Healthcare) network under Component 2 of Mission 6 of the PNRR, titled “Innovation, Research, and Digitalization of the National Healthcare System,” it is essential to prioritize technological, organizational, and professional excellence. Each network must be equipped with its own governance structure, including health professionals, capable of expressing a precise strategic vision, translating it into actionable programs (including international outreach and marketing), and being accountable for the outcome achieved for the benefit of the network and its constituent centers (PL 16). Specifically, professionals in the technical-diagnostic area can play a key role in shaping technology investment plans as part of the IRCCS reform and, more broadly, in the implementation of Investment 1.1, “Modernization of the hospital technology and digital infrastructure” across all care settings. Their specific expertise also enables them to design pilot programs for the introduction of technological innovations, such as the “managed-entry agreement” (PL 18), and to refine the hub & spoke model for the optimal redistribution of medical equipment (PL 19) (42).

Health professionals working in the area of prevention can contribute significantly to strengthening hospital infrastructure under Investment 1.2 “Towards a safe and sustainable hospital.” Their contributions include overseeing structural renovation projects (PL 20), enhancing programs and investments aimed at preventing healthcare-associated infections (PL 21), developing target training on clinical risk management (PL 22), and designing and implementing physical and psychological health surveillance programs for healthcare workers (PL 23) (41).

All these ambitious project lines can only be successfully implemented if Italian AHPs are adequately trained and supported. In this regard, it is necessary to prepare and implement a training plan (11) aimed at enhancing digital competencies across all professional profiles. This will empower healthcare workers to lead the digital transformation of the NHS and home-based care processes. Training should also extend to managerial and organizational skills, involving both formal and informal caregivers. Importantly, this education must not be limited to top NHS management but should be made a prerequisite for all healthcare professionals in coordination and organizational roles, serving as a requirement for appointments and/or renewals in such positions (PL 27) (58, 62). On the other hand, the FNO TSRM & PSTRP, through the creation of its own research center, is committed to promoting and enhancing the contribution of professionals it represents in research activities as an integral part of their practice based on evidence-based medicine (EBM). The Federation and RCs can offer valuable contributions to biomedical research within the NHS by monitoring and interpreting, in collaboration with other healthcare system stakeholders, the preventive, diagnostic, therapeutic, and care processes that define each individual’s care pathway. They can also jointly test and validate the organizational and care models and propose innovative projects and solutions to improve these processes (PL 25). This approach supports the implementation of investments 2.1 “Strengthening Biomedical Research in the NHS “and 2.2 “Development of technical, professional, digital and managerial skills of healthcare staff.”

#### 3.2.3 Application of project lines across five strategic initiatives

Each project line was concretely implemented through the proposal of five targeted initiatives:

(A) Rethinking Primary Healthcare in Community Houses (PLs 1, 3, 4, and 7).
(B) Strategically Upgrading Hospital Technology and Digital Infrastructure (PLs 17, 18, and 19).
(C) Enhancing Home Remote Assistance (PLs 10, 11, 12, and 13).
(D) Rethinking Primary Healthcare in Community Hospitals (PLs 14 and 15).
(E) Establishing an Observatory for Monitoring PNRR Implementation (PLs 24, 25, and 26).

The initiative “Rethinking Primary Healthcare in Community Houses” aims to establish an institutional and organizational framework that enables professionals from the Federation to actively participate in assessing and addressing the health and social needs of the local community where the Community Houses are located. Key objectives include:

- Implementing health education programs to promote healthy lifestyles for people of all ages, with a focus on disadvantaged populations and individuals with chronic conditions or disabilities. Examples include nutritional education, physical-motor rehabilitation, sensory therapies, and cognitive-behavioral interventions.
- Delivering educational initiatives to prevent social distress and risky behaviors.
- Applying primary, secondary, and tertiary prevention strategies targeting individuals, at-risk groups, and communities—through risk mitigation and protective interventions, both pharmacological and non-pharmacological.
- Conducting screening programs for the general population and at-risk groups, from childhood to adult age, to stratify risks and identify vulnerable individuals early.
- Developing inclusive, multicultural services accessible to all demographic groups (e.g., migrants, people of varying ages, income levels, and educational backgrounds) while promoting gender equality.
- Providing accessible “low-tech” diagnostic services.
- Facilitating continuity of care across hospital community and home setting through integrated territorial care models and multiprofessional teams.
- Promoting a data-driven culture and intervention research to validate practices and protocols.

The project “Strategically Upgrading Hospital Technology and Digital Infrastructure” focuses on a strategic modernization of hospital technological and digital systems aligned with the hub-and-spoke organizational model. It includes:

- Reassessing installed equipment in terms of quantity (what is needed), quality (technological level), and logistics (optimal distribution across facilities).
- Introducing and managing innovations that are strategically important for addressing specific health needs or enabling new treatment possibilities through technological advancement.

The project supports the continuous modernization of hospital infrastructure across specialties where FNO TSRM & PSTRP professionals operate. It aims to (a) assist decision-makers (regions and healthcare organizations) in defining standards for technology use across facility types; (b) collaborate with regional authorities to map large-scale equipment by specialty; (c) provide recommendations for identifying investment priorities; (d) Identify strategic innovations aligned with NHS and PNRR goals; (e) develop Health Technologu Assessment (HTA) protocols to guide technology implementation; (f) design training programs for professionals as users and evaluators of technology, enabling their participation in HTA committees; and (g) establish partnerships with industry stakeholders to co-develop and optimize technologies in response to specific health needs.

The initiative “Enhancing Home Remote assistance” aims to expand and enhance remote assistance services within Integrated Home Care, involving all 19 professionals represented by FNO TSRM & PSTRP. It supports the delivery of remote health and social care services at home by strengthening professional roles through remote engagement with patients and caregivers, including therapeutic agreements and training in the use of home-based technologies.

- Improving service effectiveness by supporting scheduled and repeatable home care interventions.
- Promoting continuity of care between hospital and community settings for patients in post-acute, chronic, or exacerbated phases across all age groups,
- Integrating healthcare services with municipal social and welfare services for comprehensive care.
- Leveraging ICT and digital health tools to redesign home care models tailored to the complex needs of chronic patients.

The initiative “Rethinking Primary Healthcare in Community Hospitals” (PLs 14 and 15) involves all professional categories represented by the FNO TSRM & PSTRP and focuses on defining standards for both the professional composition and technological infrastructure required for multiprofessional teams operating with community hospitals. These facilities are designed to reinforce intermediate care at the territorial level by delivering health services of medium to low clinical complexity—services that are not manageable at home but do not necessitate higher-level hospitalization. The overarching goal is to ensure more appropriate care pathways, reduce inappropriate use of emergency departments and second-level hospitalizations, and limit unnecessary reliance on specialized outpatient services. The FNO TSRM & PSTRP emphasizes that this integration must be achieved through strong collaboration between COTs, Community Houses, and the first- and second-level hospital networks. This collaboration should be supported by digitization and integration of care processes and digital platforms. In alignment with the objectives of PNRR, the project proposes to:

- Define organizational models for multiprofessional and multidisciplinary teams within community hospitals.
- Establish structural, organizational, and technological standards for territorial care in these settings.
- Develop specific diagnosis, therapeutic, and home care protocols for patients discharged from community hospitals.
- Monitor compliance with these standards at a national level.

Finally, the initiative “Establishing an Observatory for Monitoring PNRR Implementation” (PLs 24, 25, and 26) aims to establish a research center called “Studies and actions for the Innovations in Healthcare- SAPIS” to support strategic planning, implementation, and monitoring of projects proposed by the Federation and funded through PNRR resources. SAPIS research center serves as a complementary governance and supervisory body, with responsibilities that include:

- Ensuring the quality and consistency of the project implemented by supporting and coordinating the work of Federation professionals involved in the renewal of the Italian NHS’s organizational structure and the enhancement of scientific and professional accreditation.
- Providing continuous monitoring and feedback to guide corrective actions and ensure alignment with project objectives.
- Organizing and delivering training activities for professionals engaged in individual projects, thereby strengthening their capacity to contribute effectively to the transformation of the healthcare system.

**Figure 2:**
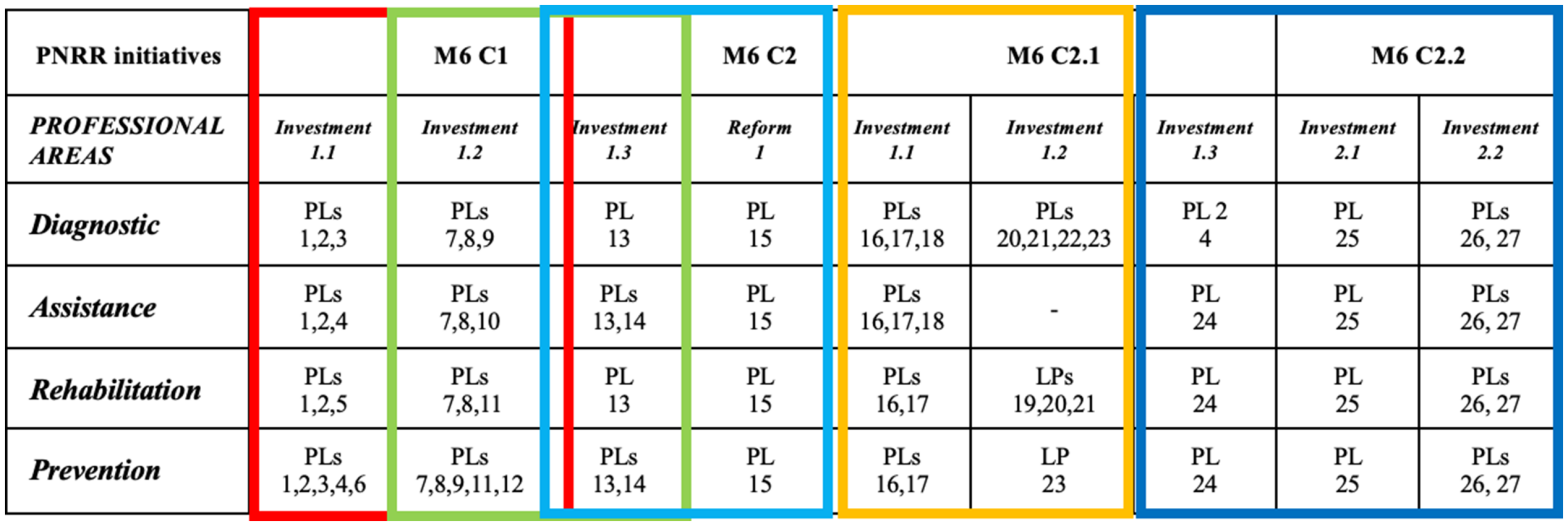
Synoptic table.

A synoptic table summarizes the PNRR goals, professional areas of the APHs belonging to FNO TSRM & PSTRP, project lines, and the five projects jointly developed by the focus group (Figure 2).

### 3.3 Position paper on “The relaunch of the Italian healthcare system. Proposals for the implementation of the National Recovery and Resilience Plan. The contribution of the FNO TSRM & PSTRP’s 19 professionals”

The final version of the position paper is titled “The relaunch of the Italian national healthcare service. Proposals for the implementation of the National Recovery and Resilience Plan. The contribution of the FNO TSRM & PSTRP’s 19 professions.” It represents the integration of all contributions from participants involved in three series of focus groups.

**Figure 3:**
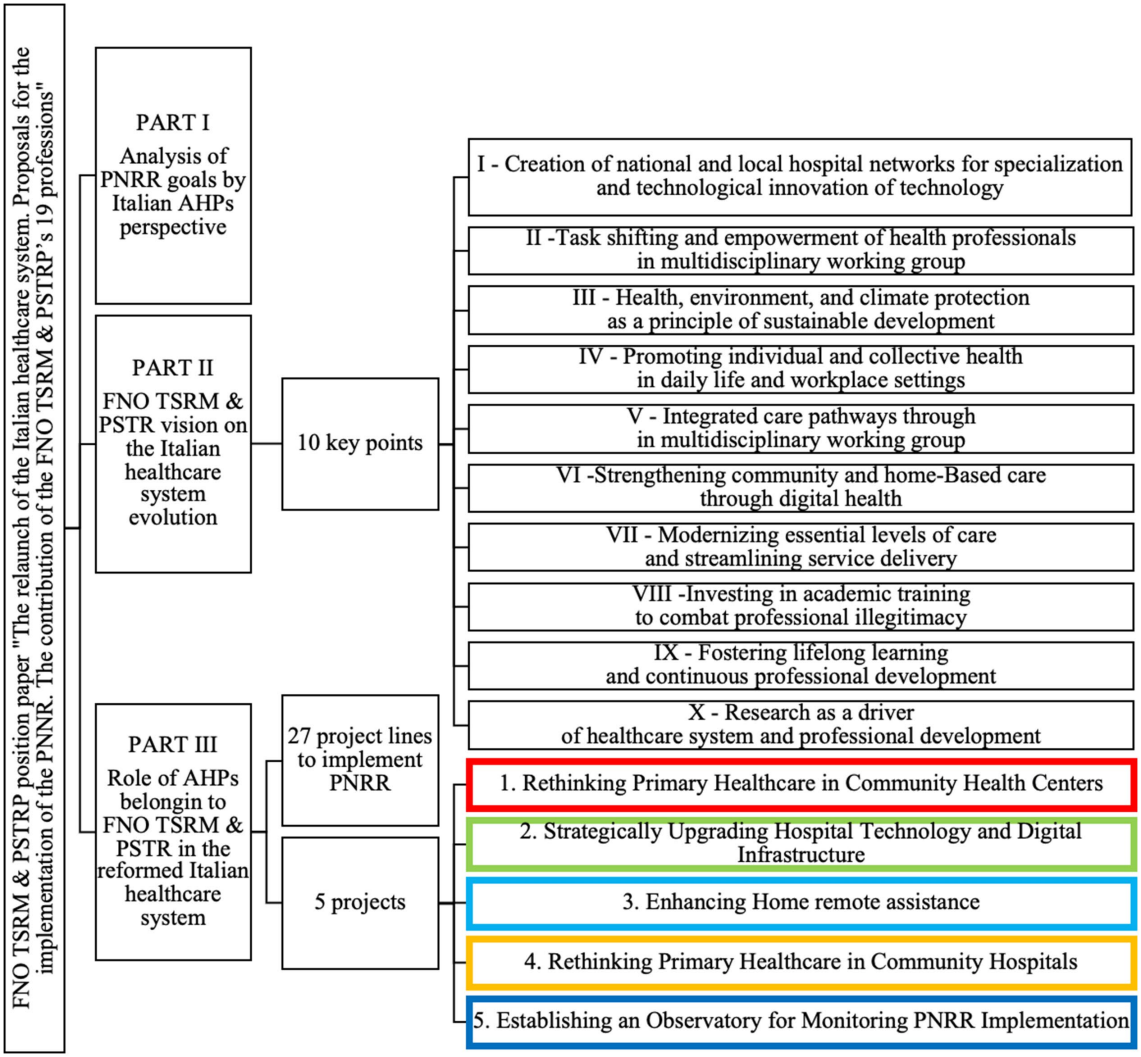
Structure of the position paper entitled “The relaunch of the Italian national healthcare service. Proposals for the implementation of the National Recovery and Resilience Plan. The contribution of the FNO TSRM & PSTRP’s 19 professionals”.

The structure of the position paper reflects the sequential steps outlined in the study protocols (Figure 3).

Part I revisits the reflections made by Research Coordinators (RCs) during the development of the requested short document. Part II provides a detailed description of the 10 key points underpinning FNO TSRM & PSTRP’s vision for the evolution of Italy’s National Health Service. Part III outlines an operational pathway consisting of 27 project lines and five projects aimed at implementing the PNRR. These initiatives emphasize an active and recognized role for Italian AHPs within a reformed healthcare system.

## 4 Conclusion

This study examines the collaborative development of a position paper titled “The relaunch of the Italian national healthcare service. Proposals for the implementation of the National Recovery and Resilience Plan. The contribution of the FNO TSRM & PSTRP’s 19 professionals.” It focuses on the ongoing reform of the Italian National Healthcare System by the 19 health professionals represented by the National Federation of Radiographers and Technical, Rehabilitation, and Prevention Health Professionals (FNO TSRM & PSTRP) in 2021.

Beginning with proposals from position papers for each professional, focus groups collected key elements to define FNO TSRM & PSTRP’s vision for the evolution of the Italian healthcare system and the role of its health professionals within this reformed framework. These contributions served as the foundation for creating a unified position paper that achieved three main objectives:

- Raising awareness about the specificities and excellence of each of the 19 health professionals represented by FNO TSRM & PSTRP.
- Clearly articulating FNO TSRM & PSTRP’s vision for reforming Italy’s National Healthcare System, emphasizing an open, multiprofessional, and inclusive approach.
- Providing operational guidance to initiate reforms in a way that maximizes benefits from the substantial investments made through PNRR resources, including both grants and loans.

By synthesizing current evidence and best practices, this study offers actionable insights for policymakers, healthcare leaders, and allied health professionals (AHPs). It aims to foster greater inclusion of these professionals in shaping a reformed Italian healthcare system grounded in multiprofessional and multidisciplinary community-based working groups, ensuring its sustainable evolution over time.

## Data Availability

The original contributions presented in the study are included in the article, further inquiries can be directed to the corresponding author.

## Footnotes

^1^Institutes for Scientific Research and Healthcare (IRCCS) (65).
